# Coloring hidden viruses

**DOI:** 10.7554/eLife.37732

**Published:** 2018-05-29

**Authors:** Marina Lusic

**Affiliations:** Center for Integrative Infectious Disease ResearchUniversity of HeidelbergHeidelbergGermany

**Keywords:** HIV-1 latency, latency reversing agents, integration sites, latency reversal, reservoirs, Human, Virus

## Abstract

An improved dual-color reporter reveals how the fate of latent HIV-1 depends on where it integrates in the human genome.

**Related research article** Battivelli E, Dahabieh MS, Abdel-Mohsen M, Svensson JP, Tojal Da Silva I, Cohn LB, Gramatica A, Deeks S, Greene WC, Pillai SK, Verdin E. 2018. Distinct chromatin functional states correlate with HIV latency reactivation in infected primary CD4^+^ T Cells. *eLife* **7**:e34655. doi: 10.7554/eLife.34655

Current antiviral therapies can suppress HIV-1 in the bloodstream to almost undetectable levels. Yet, if this therapy is interrupted, the number of viruses can start to increase again and go on to conquer the defenses of the immune system. This occurs when viruses that are in a dormant, or ‘latent’, state become reactivated. Characterized by limited gene expression and zero viral replication, latent viruses remain hidden from the immune system and are not affected by antiviral drugs, unless they are reactivated.

Latent HIV-1 is integrated within the genomes of immune cells, mainly resting CD4^+^ T cells and, to a lesser extent, macrophages (Churchill et al., 2016). Cells that represent latent reservoirs of HIV-1 are usually very rare and difficult to separate from the neighboring non-infected cells. This means that few of these cells have been available for study, which in turn has severely hampered our understanding of the mechanisms behind viral reactivation. Now, in eLife, Eric Verdin and colleagues – including Emilie Battivelli as first author – report on an improved ‘dual-color virus’ that allows cells harboring hidden latent viruses to be identified and isolated (Battivelli et al., 2018).

Over the past decade, much HIV-1 research has looked for ways to eliminate the latent viral reservoirs by first using pharmacological molecules to reverse latency in a ‘shock and kill’ approach (Deeks et al., 2012). The increased levels of gene expression in the reactivated viruses should lead to viral antigens being presented on the surface of latently infected cells. This in turn would allow the immune system to find and clear these cells and, typically, make the viruses susceptible to antiviral therapy again (Churchill et al., 2016; Margolis et al., 2016). However, this shock and kill approach only had limited success, mostly because it could not completely reactivate the virus from its latent state.

To develop more effective approaches for reactivating viruses we first need better ways to obtain resting CD4^+^ T cells that contain latent viruses in order to study them. So-called dual-color viruses – which have two fluorescent reporters under the control of different promoters – can help with this (Calvanese et al., 2013; Chavez et al., 2015). With the original version of this reporter virus (called HIV_DuoFluoI_), infected cells would glow red, making them clearly distinct from non-infected cells. If the integrated virus was active, a green fluorescent protein was also produced and the cells appeared both red and green. Latently infected cells (i.e., those with integrated yet inactive virus) could thus easily be distinguished by their pure red color.

Although this is how the tool should have worked in theory, there was room for improvement. Some of the sequences used in HIV_DuoFluoI_ could readily recombine, meaning this dual-color virus was prone to losing its reporters, which made it impossible to track reliably. Battivelli et al. – who are based at the Gladstone Institutes, UCSF, the Buck Institute for Research on Aging and other institutes across the United States, Sweden and Brazil – overcame this specific issue by changing some sequences to create an improved dual-color virus. The new version, called HIV_GKO_, contains a different green reporter (a codon-switched eGFP) under the control of the HIV-1 specific promoter. It also has an unrelated orange, rather than red, fluorescent protein (mutated Kusubira Orange) under the control of the constitutive promoter.

HIV_GKO_ allowed Battivelli et al. to examine the integration sites of latent viruses that could be reactivated and to understand how the genetic material around those sites was packaged in the nucleus (also known as the ‘chromatin context’). They could then compare these results to those from the viruses that could not be reactivated. Battivelli et al. designed their study to compare the potency of several drugs that were known to reactivate latent reservoirs via different mechanisms (Barton et al., 2016; Conrad et al., 2017; Mehla et al., 2010).

All the drugs tested showed limited reactivation unless they were used in combination. Battivelli et al. then mapped HIV-1 insertions from three different groups of infected cells. The first group contained cells with an integrated virus that was actively producing copies of itself – or productively infected cells (Figure 1). The second and third groups were non-reactivated and reactivated latently infected cells, respectively. Battivelli et al. further defined the chromatin context of the integrated viruses, and found that viruses within both productively infected cells and reactivated latently infected cells reside mainly in the active chromatin of transcribed genes and enhancers (Chen et al., 2017). Their analysis showed that viruses found in non-reactivated latently infected cells are detected within large genomic regions that interact with the dense meshwork of proteins that line the inner surface of the nucleus envelope, the nuclear lamina (Guelen et al., 2008; Marini et al., 2015). Some repressed viruses that could be reactivated were also found in the same region in reactivated latently infected cells.

**Figure 1.**
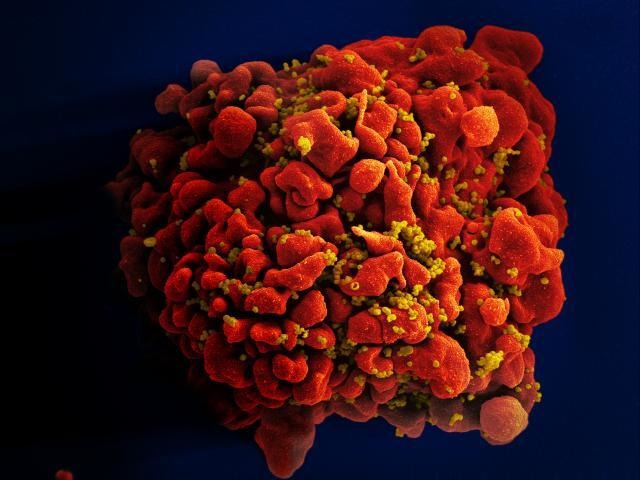
Scanning electron micrograph of an HIV-infected T cell. The human immunodeficiency virus (HIV; yellow) buds from the surface of a productively infected T cell (red).

IMAGE CREDIT: NIAID.

The fact that all the drugs tested only partially reactivated a small portion of latent viruses implies that latent reservoirs of HIV-1 are heterogeneous in nature. This finding also clearly points to the fact that transcriptional repression of HIV-1 can be influenced by the context of where it integrates in the host cell’s genome. As this is a first study where this context could be connected directly to the fate of HIV-1 infection, it becomes clear that there are many lessons to be learned about how HIV-1 explores the human genome (especially in T cells) to integrate and persist.
